# Heat shock and glycine betaine treatments partially alleviate chilling injury in banana by enhancing phenolic and sugar metabolism

**DOI:** 10.1016/j.fochx.2025.103423

**Published:** 2025-12-20

**Authors:** Nadia Niaz, Khubaib Ali, Wanfeng Hu, Siyi Pan, Robert Mugabi, Gulzar Ahmad Nayik, Noman Walayat, Mangang Wu, Isam A. Mohamed Ahmed, Guoxun Chen

**Affiliations:** a College of Food Science and Technology, Huazhong Agriculture University, Wuhan 430070, PR China; b Key Laboratory of Environment Correlative Dietology (Huazhong Agricultural University), Ministry of Education, China; c College of Food Science and Technology, Yangzhou University, Yangzhou, Jiangsu 225009, China; d Department of Food Technology and Nutrition, Makerere University, Kampala, Uganda; e Marwadi University Research Centre, Department of Microbiology, Marwadi University, Rajkot 360003, Gujarat, India; f College of Tea Science and Tea Culture, Zhejiang A&F University, 311300 Hangzhou, China; g Department of Food Science and Nutrition, College of Food and Agricultural Sciences, King Saud University, Riyadh, Saudi Arabia

**Keywords:** Bananas, Chilling injury, Postharvest alleviation, glycine betaine, Hot water, Hot air

## Abstract

Bananas are a widely consumed fruit in the world, but are highly susceptible to chilling injury below 13 °C, which reduces their quality. This study investigates the impact of hot water treatment (HWT), combined hot water and glycine betaine (HWT + GB), and hot air treatment (HAT) affects banana sugar and phenylpropanoid metabolism under cold stress, and the role of phenolics and soluble sugars in the alleviation of chilling injury (CI). The research findings showed GB + HWT treatment reduced the chilling CI index of bananas by 48 % and maintained fruit firmness. This treatment also showed increased TPC (25 %), TFC (47 %), PAL (29 %), 4CL (51 %), and C4H (50 %) activities and promoted phenolic compounds accumulation. GB + HWT treatment regulates sugar-metabolizing enzymes and balances sucrose, fructose, and glucose levels. The findings indicate that GB + HWT treatments alleviate chilling injury in bananas by stimulating phenolic and sugar metabolism, thereby enhancing fruit quality and extending shelf life.

## Highlights

• Glycine betaine plus hot water reduced banana chilling injury by 48 %.
• Treatments enhanced phenolic metabolism through increased PAL, C4H, and 4CL activities.
• Sugar metabolism enzymes (AI, SPS, SS) were positively regulated by treatments.
• Soluble sugars (sucrose, fructose, glucose) accumulated more in treated bananas.
• Combined GB + HWT treatment maintained firmness and improved postharvest fruit quality.

## 1 Introduction

Bananas rank among the most widely grown and consumed fruit globally, yet their quality and postharvest shelf life are affected by chilling damage when exposed to temperatures below 13 °C. Although initial chilling injury (CI) symptoms may not be immediately detectable, they become apparent during ripening, manifesting as peel browning, color loss, and decreased textural integrity. This degradation impairs consumer acceptance, shortens shelf life, and reduces market value (Khademi et al., 2019; Sugianti et al., 2022; Wang et al., 2022). In addition to visual deterioration, CI affects sugar and phenolic metabolism, which are critical processes for banana ripening and quality. Soluble sugars, especially sucrose, fructose, and glucose, are necessary for fruit flavor, energy supply, and membrane stability. Notably, sucrose is an important cryoprotectant, and its buildup has been associated with better chilling tolerance in a variety of fruits, including peaches and mandarins (Abidi et al., 2015; Wang et al., 2013). Furthermore, sugar metabolism affects overall fruit quality by influencing taste, texture, and oxidative stress resistance (Vimolmangkang et al., 2016). Increased soluble sugar concentrations have been linked to improved chilling tolerance in a variety of fruit cultivars, serving as osmoprotectant that assist in maintaining cellular membranes (Sevillano et al., 2009; Zhao et al., 2019; Zhao et al., 2021). In a commercial handling system, the bananas are often subjected to short-term cold-shock accidents in transit at the airport, loading of containers, and temporary storage, where the temperature can fall to 6–8 °C temporarily. Such brief exposures are not long enough to induce permanent damage but just enough to generate early CI responses, which are an appropriate model for investigating the early physiological disturbances occurring under realistic postharvest exposures.

CI in bananas is generally characterized by disturbances in phenylpropanoid metabolism, which result in excessive enzymatic browning. This happens when the polyphenol oxidase enzyme (PPO) oxidizes phenolic molecules into quinones, which then convert into melanin, resulting in tissue discoloration (Sarkar et al., 2024). Under cold stress, phenolic compounds accumulate as a defense mechanism; nevertheless, uncontrolled oxidation increases browning and quality loss (Esmaeili et al., 2017). The enzyme phenylalanine ammonia-lyase (PAL) in the phenylpropanoid pathway is a critical regulator of phenolic biosynthesis, converting phenylalanine into trans-cinnamate, a precursor for numerous phenolic compounds such as caffeic acid and chlorogenic acid (Bashmil et al., 2021). Although the impact of PAL in CI-related browning has been investigated in other fruits such as lettuce and peaches, its relevance in banana CI remains mainly unknown (Cho et al., 2016). Furthermore, peroxidase (POD) promotes oxidative browning by accelerating the oxidation of phenolic substances in response to stress (Li et al., 2021). A previous study has demonstrated that boosting phenolic and flavonoid production via treatments such as temperature conditioning and plant growth regulators like 2,4-epibrassinolide can successfully reduce CI symptoms in postharvest fruit (Islam et al., 2022). The joint heat shock and osmoprotectant synergistic effect on bananas is under consideration. Bananas have unique cold sensitivity and metabolic characteristics in relation to other climacteric fruits, especially those of phenylpropanoid and sugar. Thus, there is a need to test the synergistic effect of the combined treatment on bananas to find out whether the same synergistic effects are observed or species-specific reactions.

Postharvest methods such as heat shock treatments and osmoprotectant have shown the potential to reduce CI. Glycine betaine (GB), a naturally occurring quaternary ammonium molecule, acts as an osmoprotectant, stabilizing cellular membranes and improving plant stress tolerance. GB enhances chilling resistance in fruit, including loquat, sweet pepper, and banana, by enhancing antioxidant enzyme activity and protective metabolites like proline and phenolic compounds (Rodríguez-Roque et al., 2013; Zhang et al., 2016). Furthermore, hot water treatment (HWT) has frequently alleviated CI symptoms by increasing heat shock proteins, antioxidants, and polyamines, thus improving fruit stress tolerance (Wu et al., 2023). While the individual impacts of GB and HWT on CI have been investigated, their combined efficacy in improving sugar and phenolic metabolism in chilling-stressed bananas remains unknown. Industrially, both GB and HWT are appealing since they are food safe, low-cost, and can be used in combination with already available systems of postharvest washing and sanitation. HWT has already been used in any type of tropical fruit packing plant, and GB can be added to dipping operations without the need to have extra equipment. Knowledge of their joint impacts on bananas can thus offer a viable approach to curbing CI in the business distribution.

The goals of this research were to: (i) assess the effect of HWT, GB + HWT, and HAT on sugar and phenylpropanoid metabolism in CI-stressed bananas and (ii) explore the potential role of phenolic compounds and soluble sugars in the alleviation process of unripe and ripe bananas after chilling exposure. Understanding these pathways might lead to more effective postharvest measures for preserving banana quality, minimizing CI symptoms, and increasing storage life.

## 2 Materials and methods

### 2.1 Chemicals

The chemicals include ethanol, sodium carbonate, Folin-Ciocalteu, NaNO_2_, Al (NO_3_)_3_, NaOH, PVP, boric acid, *L*-phenylalanine, HCl, glycerol, MgCL_2_, 2-hydroxy-1-ethanethiol, PMSF, ascorbic acid, leupeptin, EDTA, CoA-SH, p-coumaric acid, ATP, methanol, formic acid, sucrose, 3,5- dinitro salicylic acid, EDTA-Na_2_, bovine serum, detassel, fructose-6-phosphate, UDPG, resorcinol, fructose, glucose, sulfuric acid, anthraquinone, and acetonitrile were acquired from Sinopharm (China). All chemicals were of analytical grade.

### 2.2 Plant material and treatments

After purchasing mature green bananas with 70 %–80 % ripeness (*Musa* spp. *Cv. ‘Cavendish’*) from Guangxia, Hubei, China, Huazhong Agricultural University examined uniform, defect-free fingers in early June.

In the preliminary analysis, 126 banana fingers were chilled at 7 °C for 2 h and separated into six groups. Four groups were tested for GB treatment (0-, 50-, 100-, and 150-mM GB in hot water (52 °C for 5 min)), and two groups received hot air (30 °C and 35 °C for 5 min). All these bananas were kept at 20 °C and 85 % RH for the next 12 d. Moreover, ripening was stimulated with ethephon (0.1 % for 1 min) and then stored for 9 d. The preliminary study results revealed that hot water (52 °C), GB mixed with hot water (100 mM GB + 52 °C), and hot air (35 °C) alleviate CI symptoms, maintain fruit quality, and prevent oxidative damage. These treatments were further analyzed for their effects on phenolic content and sugar metabolism.

105 bananas were divided into five groups for the main experiment, to assess CI alleviation after different treatments. All bananas were refrigerated for 2 h at 7 °C except group (T_0_), which was not chilled. After chilling, T_1_ bananas were treated with distilled water at 25 °C, T_2_ with hot water at 52 °C, T_3_ with 100 mM GB and hot water at 52 °C, and T_4_ with hot air at 35 °C. Each treatment lasted 5 min as shown in Table 1. Air-dried bananas were stored at 20 °C ± 1 °C and 85 % ± 3 % relative humidity for 12 days after treatments. Before further storage, all groups received 0.1 % ethephon to induce ripening. Every 3 days for 21 days, chilling damage symptoms and firmness were measured, with extra samples frozen for biochemical analysis. For reliability and accuracy, each treatment was repeated three times as indicated in the **schematic diagram.**

**Table 1:** Post-harvest treatments applied to bananas to alleviate chilling injury.

Treatment	Description of Treatment	Temperature	Duration
Normal (T0)	No chilling, no treatment	–	–
Control	Distilled water treatment	25 °C	5 min
HWT	Hot water treatment	52 °C	5 min
GB + HWT	Dipping in hot water with 100 mM GB	52 °C	5 min
HAT	Hot air treatment	35 °C	5 min

### 2.3 Chilling injury index

Chilling damage in stored bananas was visually assessed based on the extent of peel browning using a quantified grading scale as described by Tian et al. (2022). The following classification criteria were used: 0 = no browning, 1 = 1 %-20 % browning, 2 = 21 %-40 % browning, 3 = 41 %-60 % browning, 4 = 61 %-80 % browning, and 5 ≥ 81 % browning. Three biological replicates were used for this analysis.

### 2.4 Measurement of firmness

Fruit firmness was assessed with a TA- XT2i, texture analyzer (Stable Microsystems, Surrey, UK) using a 5 mm probe at a speed of 1 mm s^−1^. Four tests were conducted near the fruit's center, and the maximum force (newtons) was recorded.

### 2.5 Analysis of TPC and TFC

TPC was determined by following Ali et al. (2023) with minor changes. A sample of 1 g was homogenized in 1.2 mL of 70 % (*v*/v) ethanol and centrifuged (12,000 ×*g*, 4 °C, 30 min). Mix 0.5 mL of supernatant with 1.5 mL of Na_2_CO_3_ (7.5 %) and 3 mL of Folin-Ciocalteu reagent. A UV–Vis spectrophotometer (SpectraMAX M2, Molecular Devices, Beijing, China) was used to check absorbance at 760 nm after 1 h of dark incubation. Results were documented as grams of gallic acid kg^−1^ FW.

TFC was analyzed by using Islam et al. (2023), methodology with slight modifications. A banana sample of 0.3 g was extracted with 70 % ethanol and centrifuged (15,000 ×*g*, 4 °C, 20 min). 0.4 mL of supernatant was combined with 5 % NaNO_2_, vortexed, and incubated for 6 min. Incubation continued for 6 min after adding 0.4 mL of 10 % Al (NO_3_)_3_. After adding 2 mL of NaOH, a UV–Vis spectrophotometer (SpectraMAX M2, Molecular Devices, Beijing, China) assessed absorbance at 510 nm after 15 min. Results were presented in grams of rutin kg^−1^ FW.

### 2.6 Determination of PAL activity

The PAL activity in banana samples was measured using a modified method of Nguyen et al. (2004). A 0.3 g sample was homogenized in 1.2 mL of 0.2 mol L^−1^ boric acid buffer (pH 8.7) with 10 g L^−1^ polyvinylpyrrolidone (PVP). The homogenate was kept on ice for 1 h and centrifuged (10,000 ×*g*, 4 °C, 15 min). The supernatant containing the crude enzyme extract was collected for examination.

To perform the PAL experiment, mix 2 mL of boric acid buffer, 0.2 mL of enzyme extract, and 1 mL of 0.2 mol L^−1^ of *L*-phenylalanine. After mixing, the reaction mixture was incubated at 37 °C for 1 h. The reaction was stopped by adding 0.2 mL of 6 mol L^−1^ HCl. The absorbance was measured at 290 nm using a UV–Vis spectrophotometer (SpectraMAX M2, Molecular Devices, Beijing, China). A unit of PAL activity is the quantity of enzyme that increases absorbance by 0.01 at 290 nm per hour.

### 2.7 Analysis of cinnamic acid 4-hydrolase (C4H) activity

Modified Sun et al. (2022) methods were used to analyze C4H activity. To homogenize a 0.6 g banana sample, 3 mL of sodium phosphate buffer 50 mM, pH 7.8, containing PVP (0.15 %), glycerol (10 %), MgCl_2_ (4 mM), 2-hydroxy-1-ethanerhiol (15 mM), PMSF (1 mM), ascorbic acid (5 mM), and leupeptin (10 μM). To assess enzyme activity, the supernatant was centrifuged (12,000 ×*g*, 20 min, 4 °C). C4H activity was estimated by measuring absorbance at 340 nm, with one unit equaling 0.01 absorbance per hour.

### 2.8 Analysis of 4-coumarate-CoA ligase acid (4CL) activity

The Sun et al. (2022) method was modified to measure 4CL activity. A 0.6 g sample was mixed with 3 mL of 50 mM, pH 8 Tris-HCl buffer containing PVP (0.15 %), EDTA (5 mM), and glycerol (30 %). Samples were homogenized, centrifuged (12,000 ×*g*, 4 °C, 20 min). The reaction mixture (3 mL) contains Tris-HCl (200 mM, pH 8), MgCl_2_ (2.5 mM), CoA-SH (1 mM), 5 mM p-coumaric acid, ATP (2.5 mM), and 0.5 mL of enzyme extract. 4CL activity was measured by OD change at 333 nm, with 1 unit representing a 0.01 absorbance unit increase per hour.

### 2.9 HPLC analysis of individual phenol content

Phenolic extraction and HPLC analysis were conducted following modifications of Wang et al. (2019). Two g of banana were homogenized with 7 mL of anhydrous ethanol, incubated at 40 °C for 20 min with ultrasonic extraction, and then centrifuged (8000 ×*g*, 15 min, 4 °C), the extraction process was repeated three times, pooled, concentrated using a nitrogen blower, reconstituted in 0.5 mL of methanol, and filtered through a 0.22 μm filter. Analysis was performed on an Agilent C18 column (250 mm × 4.6 mm, 5 μm) using methanol (B) and 0.13 % formic acid aqueous solution (A) at a flow rate of 1.0 mL min^−1^, with a 10 μL injection, and detection was set at 280 nm. The gradient was as follows: 0–10 min: 10 % to 20 % B; 10–15 min, 20 % to 30 % B; 15–25 min, 30 % to 40 % B; 25–40 min, 40 % to 50 % B. Calibration curves (50–800 μg mL^−1^) were prepared from stock standard (1 mg mL^−1^) of phenolic compounds.

### 2.10 Sugar metabolism enzymes

#### 2.10.1 Acid invertase (AI) activity

The acid invertase activity was determined using the method outlined by Topcu et al. (2022). Extraction was achieved by grinding 0.1 g of banana pulp with 1.5 mL of distilled water, putting the resulting mixture in an ice bath for an hour, and then centrifuging it at 12,000 ×g for 20 min at 4 °C. The obtained supernatant was utilized for the measurement of AI enzyme activity. A reaction between 1 mL of crude enzyme solution, 2.5 mL of 6 pH PBS buffer, and 0.5 mL of sucrose solution was conducted for 30 min at 37 °C. 1 mL of the reaction solution was subsequently combined with 0.8 mL of 3,5-dinitrosalicylic acid and boiled for 5 min. Absorbance was measured at 540 nm upon cooling. The standard curve was produced using a glucose standard solution (1 mg mL^−1^).

#### 2.10.2 Sucrose phosphate synthase (SPS) activity

The SPS activity was assessed using the approach of Wang et al. (2019), with minor modifications. A tris-HCl buffer (pH 7) was made by including 100 mmol L^−1^ buffer, 5 mmol L^−1^ of MgCl_2_, 2 mmol L^−1^ of EDTA-Na_2_, polyvinylpyrrolidone, 0.2 % of bovine serum protein, 2 % ethanol, and 5 mmol L^−1^ of detassel. After the buffer preparation, 0.1 g of the sample was weighed, 0.5 mL of Tris-HCl was added, and thoroughly ground the mixture. Then, centrifuged at 10,000 ×*g* at 2 °C for approximately 20 min. The obtained supernatant was used as a crude enzyme extract for the assessment of SPS enzyme activity. A total of 0.05 mL of enzyme extract was mixed with 0.15 mL of 50 mmol L^−1^ Tris HCl (comprising Tris HCl, pH 7, 10 mmol L^−1^ MgCl_2_, 10 mmol L^−1^, 10 mmol L^−1^ fructose-6-phosphate, and 2–3 mmol L^−1^ UDPG). The mixture was incubated at 30 °C for 10 min. Subsequently, 0.05 mL of NaOH (2 mol L^−1^) was added to the reaction mixture, which was heated for 10 min. After cooling, 0.7 mL of 30 % HCl and 1 mL of 0.1 % resorcinol were added, and the mixture was maintained at 80 °C for an additional 10 min. Absorbance was measured at 480 nm after cooling. A sucrose standard curve was used for quantification.

#### 2.10.3 Sucrose synthase (SS) activity

The SS activity was determined using the same methodology as in SPS. The only distinction was that fructose (10 mmol L^−1^) was utilized instead of fructose-6-phosphate (10 mmol L^−1^). The sample's response was quantified as 1 mmol of sugar generated per hour.

### 2.11 Soluble sugar contents

The soluble sugar content was measured using the anthraquinone colorimetric method, following the procedure outlined by Fan et al. (2017) with slight modification. One gram of analytical-grade anhydrous sucrose was precisely weighed and diluted in distilled water to provide a 100 μg mL^−1^ sucrose standard solution. Subsequently, 0.5 mL of concentrated sulfuric acid was added to a 100 mL volumetric flask, and distilled water was added to reach the final volume. The mixture was thoroughly agitated. From this solution, a 10 mg mL^−1^ sucrose stock solution was obtained. To prepare the final 100 μg mL^−1^ sucrose standard solution, 1 mL of 10 mg mL^−1^ stock solution was diluted to 100 mL using distilled water.

A 0.1 g portion of the banana pulp sample was weighed and mixed with 1 mL of distilled water. The mixture was homogenized and heated in a boiling water bath for 10 min, then allowed to cool to room temperature. It was centrifuged at 8000 ×*g* for 10 min, and the supernatant was collected and diluted 40 times before analysis. For the colorimetric assay, 0.2 mL of the diluted extract was mixed with 0.1 mL (2 %) anthraquinone solution and 1 mL of concentrated sulfuric acid in a test tube. For the blank and standard samples, the extract was replaced with distilled water and the 100 μg mL^−1^ sucrose standard solution, respectively. All tubes were placed at 95 °C for 10 min, followed by cooling to room temperature. The absorbance was measured at 620 nm.

### 2.12 HPLC measurement of sucrose, fructose, and glucose

High purity standards of fructose, glucose, and sucrose were precisely weighed and dissolved in distilled water to prepare stock solutions of 1 mg mL^−1^ and further diluted to desired concentrations. Calibration curves were generated by injecting specified quantities of these standard solutions through an HPLC system (Ultimate-3000, Shanghai, China) and plotting the recorded peak regions against the respective concentrations. The linearity of the calibration curves was verified using additional standard concentrations.

The concentrations of fructose, glucose, and sucrose were assessed according to the methodology described by Wang et al. (2019). For sample analysis, 0.6 g of frozen banana flesh tissue was homogenized in 1 mL of 80 % ethanol. The mixture was centrifuged at 12,000 ×*g* for 20 min at 4 °C. The supernatant was filtered sequentially through a polyester sulfone (PES) cartridge with a 1.3 mm diameter and a 0.45 μm pore filter. Afterward, 1 mL of the filtrate was injected into the HPLC system using acetonitrile as the mobile phase at a flow rate of 1.01 mL minute^−1^. The HPLC system was equipped with a refractive index detector (RI-1530; Jasco) and an X-Bridge Amide column (250 mm × 4.6 mm) (Robusta®NH2 5 μ, Milan, Italy). Fructose, sucrose, and glucose were determined and calculated by comparing their retention times and integrated peak areas with those of the standard solutions. The final concentrations were expressed in grams per liter.

## 3 Results and discussions

### 3.1 Effect of treatments on CI

**Fig. 1:**
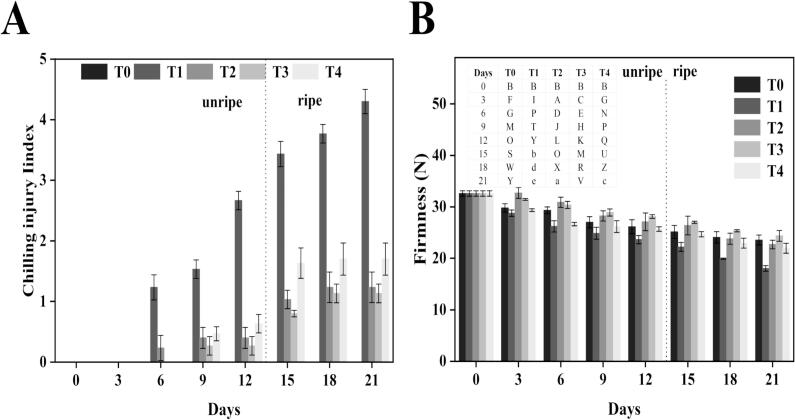
Effect of different treatments on the CI index (A) and firmness (B) of banana. Mean ± standard errors of triplicates, with vertical bars indicating the standard errors. Different letters represent significant group differences. T_0_ (no chilled, untreated), T_1_ (Chilled, treated with distilled water at 25 °C for 5 min), T_2_ (Chilled, treated with hot water at 52 °C for 5 min), T_3_ (Chilled, treated with a combination of 100 mM of glycine betaine and hot water at 52 °C for 5 min), T_4_ (Chilled, treated with hot air at 35 °C for 5 min).

CI poses a major challenge for maintaining banana quality during cold storage and shelf life (Wu et al., 2023a). In our study, bananas treated to alleviate CI showed notably less chilling damage than the control group, as shown in **Fig. S1**. Compared to control bananas, those treated for CI alleviation showed lower CI severity. After being stored at 7 °C for 2 h and then kept for 12 d, the treated groups (T_2_, T_3_, T_4_) had CI indices 37.5 %, 44.4 %, and 28.6 % lower than the control group before ripening. When ripened with 0.1 % ethephon, control bananas (T_1_) developed severe CI symptoms 21 d. In contrast, the treated groups exhibited reduced damage. Notably, T_3_ showed the most effective alleviation; at the end of the ripening process, its CI indices were 28.5 % (T_2_), 47.5 % (T_3_), and 28.6 % (T_4_) lower than those of the control group (Fig. 1**-A**). The alleviation of CI symptoms in the treated groups may be attributed to the protective effects of GB and heat shock on cellular integrity. GB has been reported to stabilize cell membranes under stress conditions by reducing lipid peroxidation and enhancing osmotic balance, as observed in peach and loquat fruit (Wang et al., 2019; Zhang et al., 2016). Furthermore, heat shock treatments are known to induce the accumulation of heat shock proteins (HSPs), which play a critical role in protecting cellular proteins and membranes from chilling-induced oxidative stress. The combined GB and hot water treatment (T_3_) likely exerts a synergistic effect by enhancing antioxidant defense systems and maintaining membrane stability, thereby significantly mitigating CI in bananas during storage and ripening. The decrease in CI was equated to an increase in the functional shelf life because treated bananas retained a reasonably good appearance and peel color over a few days under the control of the 21 d observation period.

### 3.2 Measurement of firmness

Firmness is a key indicator of fruit and vegetables (Al-Qurashi et al., 2024). In bananas, firmness generally decreases during storage before ripening; however, the rate of decrease of firmness was relatively lower in treated fruit (Fig. 1**-B**). Control samples dropped 28 % in firmness by 12 d before ripening, and 45 % by the end of 21 d of the storage period after ripening. In contrast, treated fruit maintained higher firmness levels, highlighting the effectiveness of treatments. This enhanced firmness retention in treated bananas may be attributed to the ability of heat shock and GB treatments to stabilize cell wall structures and reduce membrane permeability. GB acts as an osmoprotectant, preventing excessive water loss under stress conditions (Khademi et al., 2019), while heat shock treatments are known to activate heat shock proteins (HSPs) that protect cell wall polysaccharides and enzymes involved in softening. The observed lower weight loss in treated samples suggests reduced tissue damage and water loss during cold storage, which directly contributed to maintaining firmness. These findings align with previous studies reporting that postharvest treatments can slow the activity of cell wall-degrading enzymes such as polygalacturonase and pectin methylesterase, thereby delaying softening (Sun et al., 2022).

### 3.3 Analysis of TPC and TFC

**Fig. 2:**
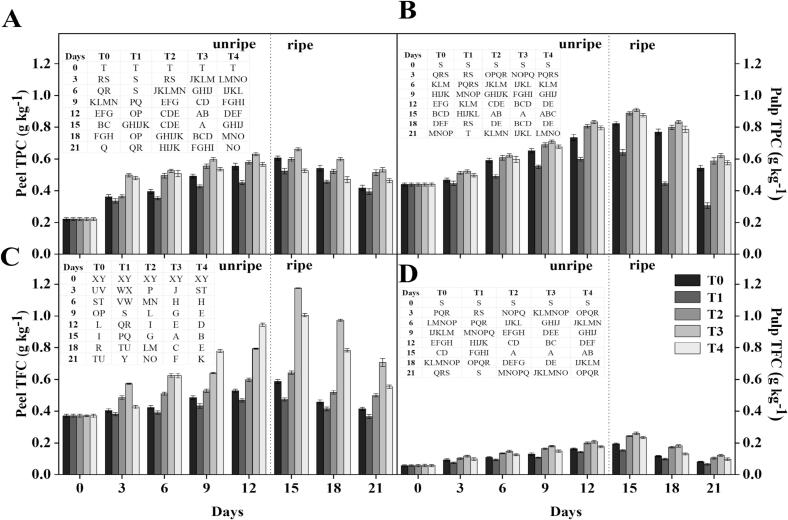
Effect of different treatments on the TPC (A-B) and TFC (C—D) of banana. Mean ± standard errors of triplicates, with vertical bars indicating the standard errors. Different letters represent significant group differences. T_0_ (no chilled, untreated), T_1_ (Chilled, treated with distilled water at 25 °C for 5 min), T_2_ (Chilled, treated with hot water at 52 °C for 5 min), T_3_ (Chilled, treated with a combination of 100 mM of glycine betaine and hot water at 52 °C for 5 min), T_4_ (Chilled, treated with hot air at 35 °C for 5 min).

The treated fruit had greater TPC and TFC levels than the control fruit **(**Fig. 2 **A, B)**. On 12 d, before ripening, T_2_ showed a 20 % increase in TPC, T_3_ showed a 28 % increase, and T_4_ had a 21 % increase. Likewise, T_2_ showed an additional 24 % increase, T_3_ showed a 25 % increase, and T_4_ showed a 16 % increase following ripening on 21 d. TPC levels in pulp tissue were similarly greater in treated fruit than in control samples. On 12 d before ripening, T_2_ showed a 25 % increase in TPC, T_3_ showed an increase of 27 %, and T_4_ showed a 23 % increase. After ripening on 21 d, T_2_ increased by 47 %, T_3_ by 49 %, and T_4_ by 45 %. Similarly, TFC levels were greater in the treated fruit peel than in the control **(**Fig. 2 **C, D)**. On 12 d before ripening, T_2_ showed a 21 % TFC increase, T_3_ showed a 40 % increase, and T_4_ showed a 49 % increase. After ripening for 21 d, T_2_ showed a rise of 28 %, T_3_ by 47 %, and T_4_ by 33 %. TFC levels in pulp tissue were likewise higher in treated fruit than in control samples. The TFC rises for T_2_, T_3,_ and T_4_ were 28 %, 31 %, and 17 %, respectively, on 12 d before ripening. After ripening on 21 d, T_2_ increased by 39 %, T_3_ by 48 %, and T_4_ by 34 %.

Research shows that heat shock and similar treatments can enhance phenolic and flavonoid levels, improving fruit tolerance to low-temperature stress (Gao et al., 2016). Our findings support this, as combining GB with HWT treatments increases TPC and TFC, boosting ROS scavenging and bolstering chilling tolerance in bananas. The enhancement of TPC and TFC in treated bananas can be attributed to the role of phenolic and flavonoid compounds as crucial antioxidants in combating reactive oxygen species (ROS) generated under chilling stress. Heat shock treatment is known to induce phenylalanine ammonia-lyase (PAL) activity, a key enzyme in the phenylpropanoid pathway, leading to increased synthesis of phenolic compounds (Gao et al., 2016). GB, on the other hand, stabilizes cellular structures and may indirectly contribute to maintaining higher antioxidant levels by protecting enzymatic activity under stress conditions. The combined GB + HWT treatment (T_3_) demonstrated a synergistic effect, resulting in the highest accumulation of phenolic and flavonoid compounds. This accumulation likely enhanced ROS scavenging capacity and reduced lipid peroxidation, contributing to improved chilling tolerance and delayed senescence in bananas (Zhang et al., 2016). Although TPC and TFC levels decreased during ripening and senescence in both control and treated fruit, the elevated baseline levels in treated samples suggest a protective role in maintaining fruit quality during storage. The increased TPC and TFC of the GB + HWT group were correlated with an increased sugar retention, suggesting that the phenolic metabolism and carbohydrate stability when exposed to cold stress could also be interacting.

### 3.4 Analysis of PAL activity

**Fig. 3:**
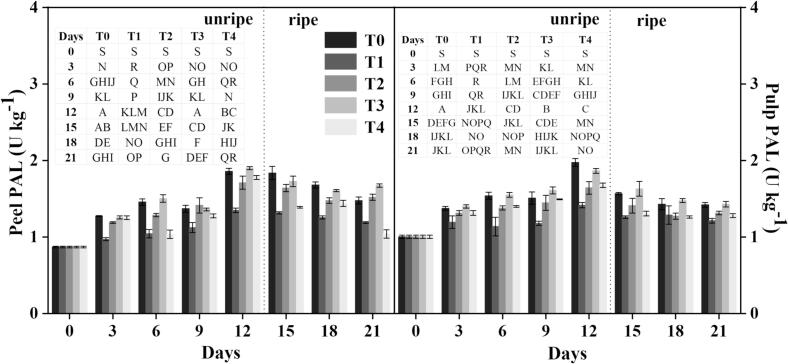
Effect of different treatments on the PAL activity of banana. Mean ± standard errors of triplicates, with vertical bars indicating the standard errors. Different letters represent significant group differences. T_0_ (no chilled, untreated), T_1_ (Chilled, treated with distilled water at 25 °C for 5 min), T_2_ (Chilled, treated with hot water at 52 °C for 5 min), T_3_ (Chilled, treated with a combination of 100 mM of glycine betaine and hot water at 52 °C for 5 min), T_4_ (Chilled, treated with hot air at 35 °C for 5 min).

Phenylpropanoids are crucial for plant defense against various stresses, and their accumulation under cold conditions is a well-known response that enhances chilling tolerance (Sharma et al., 2019). PAL is a critical enzyme in the phenylpropanoid pathway that catalyzes the deamination of phenylalanine into trans-cinnamic acid. This reaction marks the first step in synthesizing a range of phenolic compounds, lignin, and other secondary metabolites, which are vital for plant defense, structural integrity, and stress responses (Song et al., 2022). In this work, under a controlled setting and following different treatments, we assessed PAL activity in both the peel and pulp of banana samples. PAL activity increased slowly across all the samples before ripening and subsequently dropped as the fruit ripened **(**Fig. 3**)**. Especially, the treated samples often showed more PAL activity than the controls. For example, compared to the control, PAL activity was 21 %, 29 %, and 24 % greater in treatments T_2_, T_3,_ and T_4_, respectively, in peel tissues assessed on 12 d (before ripening). Treated peel samples kept high PAL activity after ripening (on 21 d), T_2_ and T_3_ showed an increase of 22 % and 29 %, respectively, and T_4_ reported a 14 % drop relative to the control. Similar patterns were seen in the pulp tissue; treated samples showed PAL activity of 14 %, 24 %, and 16 % more than control samples on 12 d, and by 21 d, the increases were 8 %, 15 %, and 5 %, respectively. Particularly in the pre-ripening stage, these findings imply that the treatments increased PAL activity, hence enhancing the production of phenolic compounds and strengthening the fruit's defensive systems. This trend is in line with other studies; for instance, investigations on apples and tomatoes have revealed that higher PAL activity is linked to higher phenolic accumulation and better fruit quality (Su et al., 2024). Studies on grapes and strawberries have also shown that stress-related PAL elevations could improve antioxidant capacity and extend shelf life (Guerrero-Chavez et al., 2015). The high PAL activity in our treated banana samples emphasizes the overall possibility of these treatments strengthening defensive responses and reducing postharvest degradation, therefore promoting better fruit quality during storage. The high level of PAL activity during the early periods of storage was supported in the initial increase of phenolic accumulation, along with the stabilizing effect of sugar, helped to delay senescence and extended shelf life.

### 3.5 C4H and 4CL activity

**Fig. 4:**
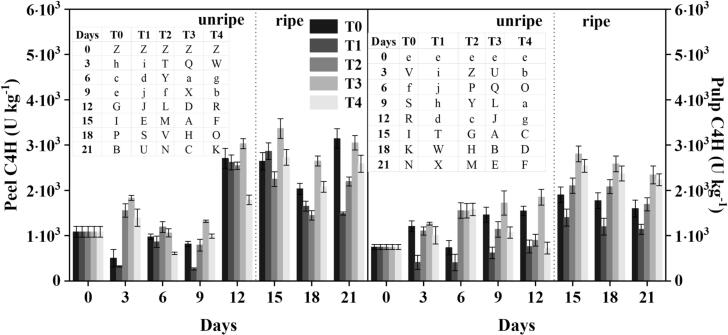
Effect of different treatments on the C4H activity of banana. Mean ± standard errors of triplicates, with vertical bars indicating the standard errors. Different letters represent significant group differences. T_0_ (no chilled, untreated), T_1_ (Chilled, treated with distilled water at 25 °C for 5 min), T_2_ (Chilled, treated with hot water at 52 °C for 5 min), T_3_ (Chilled, treated with a combination of 100 mM of glycine betaine and hot water at 52 °C for 5 min), T_4_ (Chilled, treated with hot air at 35 °C for 5 min).

Lignin, a complex aromatic polymer, is produced by the phenylpropanoid pathway (PPP), which includes enzymes such as C4H and 4CL (Sun et al., 2022). The treated groups showed greater C4H and 4CL activity than the controls, resulting in increased phenolic and flavonoid formation. On 12 d, before ripening, C4H activity in the T_3_ group was 19 % greater, whereas T_2_ and T_4_ exhibited lower activity by 4 % and 44 %, respectively, when compared to the control **(**Fig. 4**)**. After ripening on 21 d, C4H activity increased by 31 % (T_2_), 50 % (T_3_), and 43 % (T_4_), indicating that the treatments increased C4H activity, resulting in higher phenolic and flavonoid production in treated samples. Similarly, banana pulp exhibited enhanced C4H activity. On 12 d, T_2_ and T_3_ samples had 15 % and 57 % greater activity than the control, respectively, whereas T_4_ showed a 6 % reduction. By 21 d, C4H activity had risen by 31 % (T_2_), 50 % (T_3_), and 47 % (T_4_) as compared to controls.

**Fig. 5:**
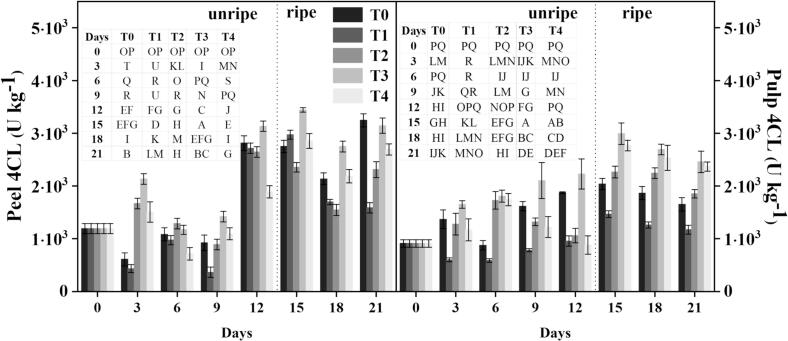
Effect of different treatments on the 4CL activity of banana. Mean ± standard errors of triplicates, with vertical bars indicating the standard errors. Different letters represent significant group differences. T_0_ (no chilled, untreated), T_1_ (Chilled, treated with distilled water at 25 °C for 5 min), T_2_ (Chilled, treated with hot water at 52 °C for 5 min), T_3_ (Chilled, treated with a combination of 100 mM of glycine betaine and hot water at 52 °C for 5 min), T_4_ (Chilled, treated with hot air at 35 °C for 5 min).

4CL enzyme activity in banana peels responded differently to treatment over time **(**Fig. 5**)**. Before ripening, on 12 d, 4CL activity in the T_3_ group was considerably higher than in the control group by 12 %, but the T_2_ and T_4_ groups had declines of 4 % and 42 %, respectively. On 21 d following ripening, 4CL activity in treated samples increased by 30 % (T_2_), 51 % (T_3_), and 40 % (T_4_). These findings suggested that these treatments increased 4CL activity over time, which may have contributed to increased flavonoid and phenolic formation in the treated samples. On 12 d, 4CL activity in T_2_ and T_3_ rose by 11 % and 56 % respectively, compared to the control, whereas T_4_ exhibited an 8 % decrease. On 21 d after ripening, 4CL activity in treated groups rose by 36 % (T_2_), 51 % (T_3_), and 49 % (T_4_) compared to control samples. These findings showed that our treatments, particularly T_3_, considerably promoted the synthesis of phenol and flavonoid contents due to the increased activity of 4CL. According to Sun et al. (2022), the cold stress conditions caused increased synthesis of these bioactive chemicals, which led to the establishment of a defensive mechanism, resulting in enhanced banana quality.

Methyl jasmonate (MeJA) can increase C4H and 4CL activity, resulting in increased phenol and flavonoid production in fresh potatoes (Zhou et al., 2019) and kiwi fruit (Li et al., 2022). The main enzymes involved in the PPP (metabolic pathway in plants) are PAL, 4CL, and C4H. PAL converts *L*-phenylalanine into trans-cinnamic acid, followed by C4H, which produces p-coumaric acid, caffeic acid, and ferulic acid (phenolic intermediates) by converting cinnamic acid (Bashmil et al., 2021). These findings demonstrate the ability of our methods to change the PPP, hence boosting the plant's antioxidant capability under stressful circumstances. The increase in PAL, C4H and 4CL aids the robust activation of phenylpropanoids in treated fruit and adds to the resultant responses of sugar metabolism, propose a coordinated defense system.

### 3.6 HPLC analysis of individual phenol content

In this work, we analyzed individual phenolic compounds like gallocatechin, epicatechin, quercetin, luteolin, and chlorogenic acid. Our data showed that these compounds tend to rise until 12 d before ripening, then either fluctuate or drop in both control and GB + HWT-treated samples. Crucially, compared to control samples, the treated samples often showed increased phenolic levels. For instance, in the peel, quercetin reached 112.6 mg kg^−1^ on the 15 d after ripening in treated fruit, compared to 108.7 mg kg^−1^ in the control. Furthermore, treated samples showed 134.1 mg kg^−1^ of luteolin on 12 d before ripening, whereas the control showed just 120.2 mg kg^−1^. In the peel, similar patterns were observed for gallocatechin (197.4 mg kg^−1^), epicatechin (110.1 mg kg^−1^), and chlorogenic acid (18.4 mg kg^−1^). In the pulp, the impact was considerably more noticeable. Compared to the control group, the treated banana had greater amounts of quercetin (124.6 mg kg^−1^), luteolin (300.9 mg kg^−1^), chlorogenic acid (39.7 mg kg^−1^), gallocatechin (695.1 mg kg^−1^), and epicatechin (191.4 mg kg^−1^), as shown in Table 2.

**Table 2:** Change in the levels of individual phenolic compounds in banana samples following GB + HWT treatment. Data are presented as mean ± standard error from three independent assays. Different letter (a-o) indicates the difference with in the treatments.

Phenolic compound (mg kg^−1^)	Quercetin		Luteolin		Chlorogenic acid		Gallocatechin		Epicatechin	
**Days**	**Peel tissue**									
	**Control**	**GB + HWT**	**Control**	**GB + HWT**	**Control**	**GB + HWT**	**Control**	**GB + HWT**	**Control**	**GB + HWT**
**0**	108.0 ± 2.9^e^	108.0 ± 2.9^e^	40.8 ± 1.1^k^	40.5 ± 1.6^k^	6.0 ± 0.2^g^	6.0 ± 0.2^g^	48.4 ± 0.8^i^	48.4 ± 0.8^i^	24.3 ± 0.6^l^	24.3 ± 0.6^l^
**3**	111.4 ± 1.6^abcd^	113.0 ± 1.1^a^	62.6 ± 1.6^j^	85.5 ± 1.7^g^	8.1 ± 0.4^f^	12.1 ± 0.4^c^	87.3 ± 1.7^f^	129.1 ± 1.1^b^	32.4 ± 0.4^j^	31.4 ± 0.4^k^
**6**	110.1 ± 1.1^abcde^	111.7 ± 2.1^abc^	80.4 ± 1.3^i^	82.5 ± 1.5^hi^	4.3 ± 0.4^hi^	8.2 ± 0.1^f^	25.6 ± 1.6^l^	49.1 ± 1.0^i^	13.4 ± 0.4^n^	77.3 ± 0.3^f^
**9**	108.4 ± 1.7^de^	110.8 ± 2.2^abcde^	83.5 ± 1.5^gh^	98.3 ± 1.7^f^	4.2 ± 0.3^i^	4.8 ± 0.3^h^	34.2 ± 1.1^j^	93.2 ± 1.1^e^	22.1 ± 0.2^m^	91.4 ± 0.6^c^
**12**	109.2 ± 1.9^cde^	111.2 ± 1.7^abcde^	120.2 ± 2.0^c^	134.1 ± 2.1^b^	12.4 ± 0.6^c^	18.4 ± 0.4^a^	31.6 ± 0.7^k^	53.8 ± 1.6^h^	32.7 ± 0.4^j^	84.3 ± 0.3^e^
**15**	108.7 ± 1.1^cde^	112.4 ± 2.3^ab^	101.8 ± 1.9^e^	112.9 ± 2.0^d^	8.3 ± 0.4^f^	11.2 ± 0.1^d^	111.9 ± 1.1^d^	197.4 ± 1.9^a^	40.7 ± 0.3^h^	110.1 ± 1.0^a^
**18**	109.3 ± 1.5^bcde^	109.4 ± 1.5^bcde^	103.3 ± 1.2^e^	137.5 ± 1.5^a^	5.8 ± 0.5^g^	13.5 ± 0.4^b^	56.4 ± 1.5^g^	121.4 ± 1.3^c^	36.8 ± 0.2^i^	43.7 ± 0.1^g^
**21**	108.7 ± 2.1^cde^	108.6 ± 1.5^cde^	98.0 ± 1.1^f^	117.5 ± 1.4^c^	8.9 ± 0.2^e^	12.2 ± 0.1^c^	32.1 ± 1.1j^k^	86.4 ± 1.3^f^	86.2 ± 0.4^d^	100.2 ± 1.1^b^
	**Pulp tissue**									
**0**	114.6 ± 3.8^c^	114.6 ± 3.8^c^	68.2 ± 2.0^k^	68.2 ± 2.0^k^	10.3 ± 2.2^h^	10.3 ± 2.2^h^	123.8 ± 3.1^o^	123.8 ± 3.1^o^	52.9 ± 4.5^j^	52.9 ± 4.5^j^
**3**	121.0 ± 1.9^b^	126.7 ± 1.6^a^	103.5 ± 1.8^i^	124.5 ± 1.6^fg^	27.6 ± 0.4^b^	40.3 ± 0.3^a^	374.6 ± 2.5^h^	134.3 ± 2.2^n^	97.9 ± 0.9^h^	107.3 ± 1.2^g^
**6**	111.3 ± 1.8^cdef^	121.2 ± 1.0^b^	99.2 ± 1.1^j^	122.1 ± 2.0^g^	13.5 ± 0.4^g^	16.4 ± 0.4^f^	307.7 ± 2.1^i^	393.6 ± 2.4^g^	109.3 ± 1.4^fg^	112.6 ± 1.4^f^
**9**	108.3 ± 1.1f^g^	110.9 ± 2.0^defg^	118.5 ± 1.5^h^	140.6 ± 2.4^d^	12.7 ± 0.3^g^	17.2 ± 0.4^f^	263.2 ± 1.9^k^	668.3 ± 4.2^b^	111.7 ± 1.1^f^	133.3 ± 1.0^d^
**12**	121.0 ± 1.0^b^	124.6 ± 1.6^a^	140.4 ± 1.6^d^	154.9 ± 1.7^c^	18.7 ± 0.1^e^	21.5 ± 0.3^d^	185.5 ± 1.5^m^	455.2 ± 2.0^e^	128.6 ± 1.5^e^	165.4 ± 1.2^b^
**15**	107.5 ± 1.5^g^	113.0 ± 1.0^cd^	121.5 ± 1.5 g^h^	286.2 ± 2.0^b^	28.7 ± 0.3^b^	39.7 ± 0.4^a^	202.3 ± 2.0^l^	489.4 ± 2.0^c^	162.7 ± 2.0^b^	191.4 ± 1.1^a^
**18**	114.5 ± 2.5^c^	110.7 ± 1.1^defg^	126.9 ± 1.9^f^	300.9 ± 2.6^a^	11.1 ± 0.4^h^	21.3 ± 0.2^d^	475.1 ± 3.0^d^	695.1 ± 2.1^a^	76.8 ± 1.9^i^	96.2 ± 1.0^h^
**21**	109.5 ± 1.4^efg^	112.8 ± 1.2^cde^	122.4 ± 2.0^g^	136.6 ± 1.5^e^	10.6 ± 0.2^h^	23.2 ± 0.4^c^	291.0 ± 2.1^j^	421.6 ± 2.5^f^	46.6 ± 0.2^k^	151.4 ± 1.6^c^

These results imply that the fruit responds to cold stress by increasing phenolic content up to 12 d before ripening and that GB + HWT accelerates phenolic compound metabolism. By scavenging ROS, the increased concentration of these molecules most likely serves as a protective function by lowering membrane peroxidation and damage linked with chilling injury. On the other hand, the reduction in phenolic content seen later in storage in both control and treated fruit is most likely the result of the ripening and senescence process, in which phenolic compounds act as antioxidants. Our results generally show that GB + HWT treatments can improve phenolic formation in bananas during the early phase of cold storage, therefore providing a possible approach to reducing CI and maintaining fruit quality. The observed increase in individual phenolic compounds such as quercetin, luteolin, and gallocatechin in treated bananas highlights the critical role of these metabolites in strengthening antioxidant defenses under chilling stress. Phenolic compounds are known to neutralize ROS, reduce lipid peroxidation, and reinforce cell wall integrity, which collectively alleviate chilling injury symptoms. The GB + HWT treatment likely activates phenylpropanoid metabolism more effectively than cold stress alone, promoting higher phenolic accumulation during the early storage phase. This mechanism aligns with previous findings where postharvest treatments such as epibrassinolide or low-temperature conditioning enhanced phenolic biosynthesis, contributing to better chilling tolerance in fruits like peaches and grapefruit (Aispuro-Hernández et al., 2019; Gao et al., 2016). The subsequent decline in phenolic content during ripening is consistent with their antioxidant function, as these compounds are utilized to mitigate oxidative stress during the senescence process.

### 3.7 Sugar metabolism enzymes

#### 3.7.1 Acid invertase (AI) activity

**Fig. 6:**
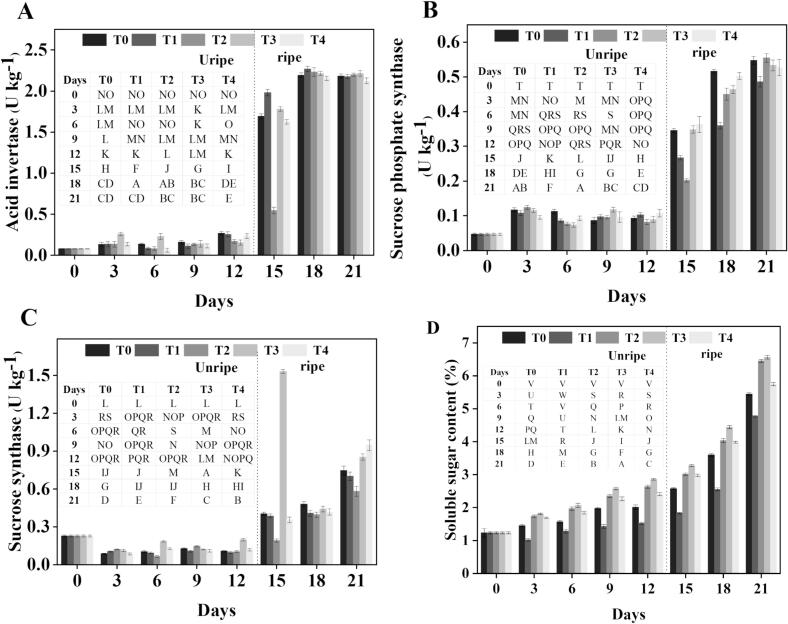
Effect of different treatments on the AI activity (A), SPS activity (B), SS activity (C), and soluble sugar contents (D) of banana. Mean ± standard errors of triplicates, with vertical bars indicating the standard errors. Different letters represent significant group differences. T_0_ (no chilled, untreated), T_1_ (Chilled, treated with distilled water at 25 °C for 5 min), T_2_ (Chilled, treated with hot water at 52 °C for 5 min), T_3_ (Chilled, treated with a combination of 100 mM of glycine betaine and hot water at 52 °C for 5 min), T_4_ (Chilled, treated with hot air at 35 °C for 5 min).

AI is vital for banana ripening since it breaks down sucrose into glucose and fructose, directly altering the fruit's sweetness and overall quality. Its activity serves a key role in maintaining the equilibrium between sucrose and its monosaccharide derivatives during ripening (Topcu et al., 2022). In our investigation, AI activity fluctuates in treated and controlled banana samples during the storage duration **(**Fig. 6**-A)**. Especially, treated samples usually showed more activity of AI than the control. In particular, the activity of AI in treatments T_2_, T_3,_ and T_4_ was 34 %, 39 %, and 9 % lower than in the control, respectively, on 12 d before ripening. On 21 d, T_2_ and T_3_ showed higher activity levels, with increases of 9 % and 15 % over the control group, while T_4_ showed a 21 % decrease in AI activity relative to the control group. Our findings coincide with the widespread knowledge that AI activity is a major factor influencing sucrose metabolism during fruit ripening when compared to early published studies. The observed fluctuations in AI activity suggest that GB and heat shock treatments modulate sucrose metabolism differently across storage stages. The initially lower AI activity in treated samples before ripening may help maintain higher sucrose reserves during cold storage, which could be advantageous in delaying senescence. After ripening, the increased AI activity in T_2_ and T_3_ likely contributes to accelerated sucrose hydrolysis into glucose and fructose, enhancing sweetness and improving fruit quality. This dual-phase modulation aligns with the role of AI in balancing sugar metabolism under stress conditions and during ripening, as reported in other fruits (Borsani et al., 2009; Zaharah et al., 2013).

#### 3.7.2 Sucrose phosphate synthase (SPS) activity

SPS is a critical enzyme in banana ripening, promoting the conversion of starch into sucrose, hence boosting the fruit's sweetness and flavor (Cordenunsi & Lajolo, 1995). Our investigation tracked SPS activity in control and treated banana samples during storage. Treated samples consistently displayed greater SPS activity compared to controls, showing that the applied treatments considerably impacted this enzyme function **(**Fig. 6**-B)**. On 12 d before ripening, SPS activity in treatments T_2_ and T_3_ was 26 % and 15 % lower than the control, respectively, while T_4_ exhibited a 5 % increase. Relative to the control, SPS activity levels in T_2_, T_3,_ and T_4_ were raised by 12 %, 9 %, and 8 %, respectively, post-ripening on 21 d. Our results coincide with the widespread knowledge that SPS activity is a crucial component in sucrose formation during fruit ripening when compared to earlier published investigations.

The modulation of SPS activity by GB and heat shock treatments suggests a possible role in optimizing sugar metabolism under cold storage stress. The lower SPS activity in T_2_ and T_3_ before ripening may contribute to slower sucrose accumulation during storage, which could help maintain fruit firmness and delay premature ripening. However, the increased SPS activity observed post-ripening in treated fruit indicates enhanced conversion of starch to sucrose during ethylene-induced ripening, improving sweetness and flavor. This pattern supports previous findings that postharvest treatments can fine-tune carbohydrate metabolism, balancing storage stability with desirable ripening characteristics (Cordenunsi & Lajolo, 1995; Do Nascimento et al., 1997).

#### 3.7.3 Sucrose synthase (SS) activity

SS helps both the synthesis and breakdown of sucrose in carbohydrate metabolism. During ripening, SS activity is critical in bananas as it controls the conversion of sucrose into other sugars and starch, therefore influencing the sweetness, texture, and general quality of the fruit (Cordenunsi & Lajolo, 1995). Throughout the storage period, we observed a rising trend in SS activity in both control and treated samples **(**Fig. 6**-C)**. In particular, treated samples showed consistently greater SS activity than the control group, suggesting that the applied treatments improved this enzymatic activity. On 12 d before ripening, SS activity in treated samples T_2_, T_3,_ and T_4_ was 8 %, 52 %, and 20 % greater, respectively, as compared to the control. On 21 d after ripening, this tendency persisted; T_2_, T_3,_ and T_4_ showed increases of 21 %, 31 %, and 49 %, respectively, over the control. These results imply that the applied treatments increase SS activity, which could improve sucrose metabolism and, thus, fruit quality during ripening. The increased SS activity in treated bananas suggests that GB and heat shock treatments enhance sucrose metabolism during storage and ripening. Higher SS activity likely facilitates balanced sucrose turnover, contributing to improved sugar composition and fruit quality. The pronounced increase in T_3_ indicates a synergistic effect of GB and HWT in maintaining carbohydrate metabolism under cold stress. These findings are consistent with previous reports showing that modulating SS activity can influence chilling tolerance and sugar profiles in postharvest fruit (Satekge & Magwaza, 2020). The ability of these treatments to sustain SS activity post-ripening may also support optimal sweetness and texture, which are critical for consumer acceptance.

### 3.8 Soluble sugar contents

Previous studies have indicated that postharvest interventions can affect sugar metabolism either by controlling sugar transit and storage mechanisms or by encouraging the enzymatic conversion of starch to sugar (Fan et al., 2017). Over the storage period, our investigation found a constant rise in soluble sugar concentration in both treated and control banana samples. However, the treated samples consistently accumulated more soluble sugars than the control group, suggesting that the postharvest treatments positively influenced sugar metabolism. Just before ripening, on 12 d, the soluble sugar concentration in the treated samples (T_2_, T_3,_ and T_4_) was 42 %, 47 %, and 37 % greater, respectively, than the control group. This tendency continued even on 21 d following ripening; the treated samples revealed noticeably greater sugars during storage. T_2_, T_3,_ and T_4_ specifically showed increases of 26 %, 27 %, and 17 %, respectively, over the control. These results imply that the treatments either reduced sugar breakdown or actively encouraged sugar biosynthesis, therefore improving the sweetness levels and improving the general fruit quality. When placed in the context of previous studies, our results align with existing literature that demonstrates the positive impact of postharvest treatments on sugar accumulation in fruit. Fan et al. (2017) found that salicylic acid treatment in strawberries raised sugar levels, which were linked to delayed senescence and enhanced fruit quality. Zhang et al. (2021) discovered that treated peaches had higher sugar levels than control ones, hence improving the storage retention of sweetness and texture. Although our study supports the idea that postharvest treatments can increase sugar content, the obvious rise in sugar seen, especially in T_3_ (47 % higher than the control before ripening), points to a more marked impact of the treatments we used on sugar metabolism. This indicates the important role of postharvest treatments in optimizing sugar accumulation, ultimately improving both the shelf life and quality of bananas. The increased levels of soluble sugar in the combined treatment did not only increase sweetness but it also acted as cryoprotectants, which helped not only in reducing CI but also increased shelf-life performance than the control.

### 3.9 HPLC measurement of sucrose, fructose, and glucose

Soluble sugars, particularly sucrose, fructose, and glucose, are fundamental to plant metabolism and structure at both the cell and whole organism levels (Wang et al., 2019). These sugars are not only essential components of energy metabolism under stress but also actively defend plants against hostile environments like drought, cold, and salt (Cordenunsi-Lysenko et al., 2019). Acting as potent antioxidants, they help protect fruit cells from oxidative damage induced by cold stress. Particularly, sucrose is known as a crucial cryoprotectant that protects plants from the negative consequences of chilling damage (Villanueva et al., 2019).

**Fig. 7:**
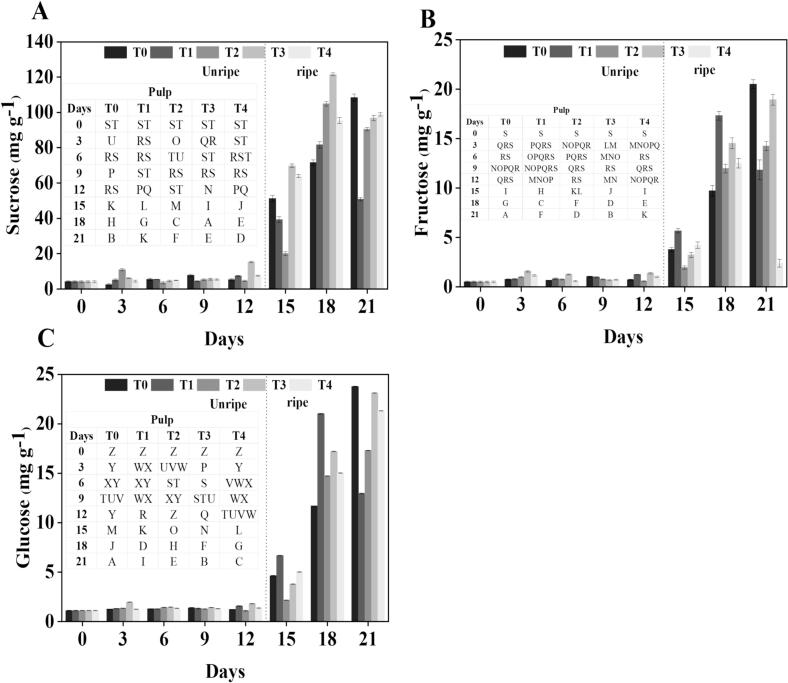
Effect of different treatments on the sucrose (A), fructose (B), and Glucose (C) of banana. Mean ± standard errors of triplicates, with vertical bars indicating the standard errors. Different letters represent significant group differences. T_0_ (no chilled, untreated), T_1_ (Chilled, treated with distilled water at 25 °C for 5 min), T_2_ (Chilled, treated with hot water at 52 °C for 5 min), T_3_ (Chilled, treated with a combination of 100 mM of glycine betaine and hot water at 52 °C for 5 min), T_4_ (Chilled, treated with hot air at 35 °C for 5 min.

Sucrose is a vital disaccharide formed from glucose and fructose and is a major determinant of banana sweetness and overall quality. In this work, sucrose concentration revealed a rising tendency in both treated and control samples under storage **(**Fig. 7**-A)**. Especially on 12 d before ripening, treatment T_2_ showed a sucrose content 60 % less than that in the control; conversely, T_3_ and T_4_ showed 25 % and 3 % greater amounts, correspondingly. On 21 d after ripening, sucrose levels in T_2_, T_3,_ and T_4_ were 44 %, 47 %, and 49 % greater than the control group. While consistently increased levels in T_3_ and T_4_ indicate that these treatments may improve sucrose synthesis by reducing its breakdown, the originally lower sucrose content in T_2_ suggests a delayed beginning of sucrose accumulation.

Fructose, a monosaccharide produced from sucrose hydrolysis, contributes to the flavor and sweetness of bananas **(**Fig. 7**-B)**. Our HPLC study found that fructose content responds differently across the treatments. Whereas T_2_ and T_4_ exhibited 54 % and 18 % lower levels, respectively, T_3_ revealed an 11 % rise in fructose relative to control on 12 d before ripening. On 21 d after ripening, fructose concentration in T_2_ and T_3_ rose by 20 % and 60 % relative to the control, and T_4_ dropped by 80 %. While the notable drop in T_4_ could indicate a change in metabolic pathways that limits fructose accumulation, the consistent rise in T_3_ suggests that this treatment essentially promotes the conversion of sucrose into fructose, a result consistent with the observation of (Zhao et al., 2019).

Glucose is a fundamental monosaccharide involved in energy metabolism and contributes to fruit sweetness. In our analysis, in every sample, glucose level rose gradually during storage **(**Fig. 7**-C)**. T_3_ noted a 13 % greater glucose content than the control on 12 d before ripening. T_2_ and T_4_ reported 43 % and 16 % lower levels, respectively. By 21 d following ripening, T_2_, T_3,_ and T_4_ risen by 25 %, 50 % and 39 %. The consistently higher glucose accumulation in T_3_ may be due to an enhanced breakdown of starch or sucrose, thereby contributing to improved sweetness as reported by Shiga et al. (2011). The initial reduced levels seen in T_2_ and T_4_ were followed by a notable rise, pointing to a delayed response in glucose metabolism, most likely resulting from distinct regulated mechanisms influencing starch breakdown under various treatments.

Higher sucrose levels have been connected in many studies to increased cold resistance. For example, Wang et al. (2019) demonstrated that increased sucrose enhanced chilling tolerance in peach fruit and assisted in providing membrane stability. Likewise, Yu et al. (2016) found that treatments including hot air and methyl jasmonate raised sugar concentration in peaches, corresponding with improved chilling tolerance, and similar findings were recorded in mandarin fruit following heat treatment (Ghasemnezhad et al., 2008). Enhanced chilling tolerance was linked to bananas treated with GB + HWT, showing greater sucrose levels than controls in our research. Interestingly, sucrose levels were constant, whereas fructose and glucose amounts changed gradually after storage. This trend could result from other carbohydrates, including starch breaking down into glucose and maybe sorbitol being converted into fructose and glucose (Shiga et al., 2011). These findings imply that GB helps to promote sucrose accumulation, hence enhancing membrane stability and changing important signal cascades. Therefore, preservation of increased sucrose levels by GB + HWT treatment may provide another method by which chilling damage is reduced in banana fruit.

## 4 Conclusion

This study systematically investigated the effects of HWT, combined GB + HWT, and HAT treatments on chilling-injured bananas. The results demonstrated that these treatments, especially GB + HWT, can notably reduce the CI index of bananas while preserving fruit firmness. At the physiological and biochemical level, GB + HWT treatment promoted the accumulation of soluble sugars, increased the content of total phenols and flavonoids, enhanced the activity of key enzymes in the phenylpropanoid metabolism pathways (PAL, C4H, and 4CL), and promoted the synthesis of phenolic compounds. Additionally, this treatment regulates the activity of sugar metabolism enzymes (AI, SPS, and SS), optimizing the metabolism of sucrose, fructose, and glucose. These results suggest that GB + HWT treatment alleviates banana chilling injury and improves fruit quality by enhancing phenolic and sugar metabolism. This study provides a novel approach to the postharvest processing of bananas, potentially reducing economic losses due to cold damage and promoting the sustainable development of the banana industry. Future research could delve deeper into the molecular mechanism underlying the GB + HWT treatment and explore its potential application in other fruits and vegetables.

## CRediT authorship contribution statement

**Nadia Niaz:** Writing – review & editing, Validation, Software, Methodology, Investigation, Conceptualization. **Khubaib Ali:** Writing – review & editing, Software, Investigation, Formal analysis. **Wanfeng Hu:** Writing – review & editing, Writing – original draft, Formal analysis, Data curation, Conceptualization. **Siyi Pan:** Writing – review & editing, Software, Funding acquisition, Formal analysis, Data curation. **Robert Mugabi:** Writing – review & editing, Software, Investigation, Formal analysis, Data curation. **Gulzar Ahmad Nayik:** Writing – review & editing, Visualization, Software, Resources, Formal analysis, Data curation. **Noman Walayat:** Writing – review & editing, Writing – original draft, Resources, Investigation, Formal analysis, Data curation. **Mangang Wu:** Writing – review & editing, Validation, Methodology, Investigation, Formal analysis, Data curation, Conceptualization. **Isam A. Mohamed Ahmed:** Writing – review & editing, Software, Methodology, Funding acquisition, Formal analysis, Data curation. **Guoxun Chen:** Writing – review & editing, Supervision, Software, Methodology, Formal analysis, Data curation.

## Funding

This study was financially supported by China's Central Government's Local Science and Technology Development Fund Projects **(**grant number 236Z7102G). The authors extend their appreciation to the Ongoing Research Funding program (ORF-2026-1074), King Saud University, Riyadh, Saudi Arabia.

## Declaration of competing interest

The authors declare that they have no known competing financial interests or personal relationships that could have appeared to influence the work reported in this paper.

## Data Availability

Data will be made available on request.

## Appendix A Supplementary data

Supplementary material Image 1
