# Supplementary material for: Heat shock and glycine betaine treatments partially alleviate chilling injury in banana by enhancing phenolic and sugar metabolism
Source: Food Chem X. 2025 Dec 20;33:103423. doi: 10.1016/j.fochx.2025.103423 (PMC12807840; doi:10.1016/j.fochx.2025.103423)
Supplement: Supplementary file 1 — Supplementary material [file mmc1.docx]

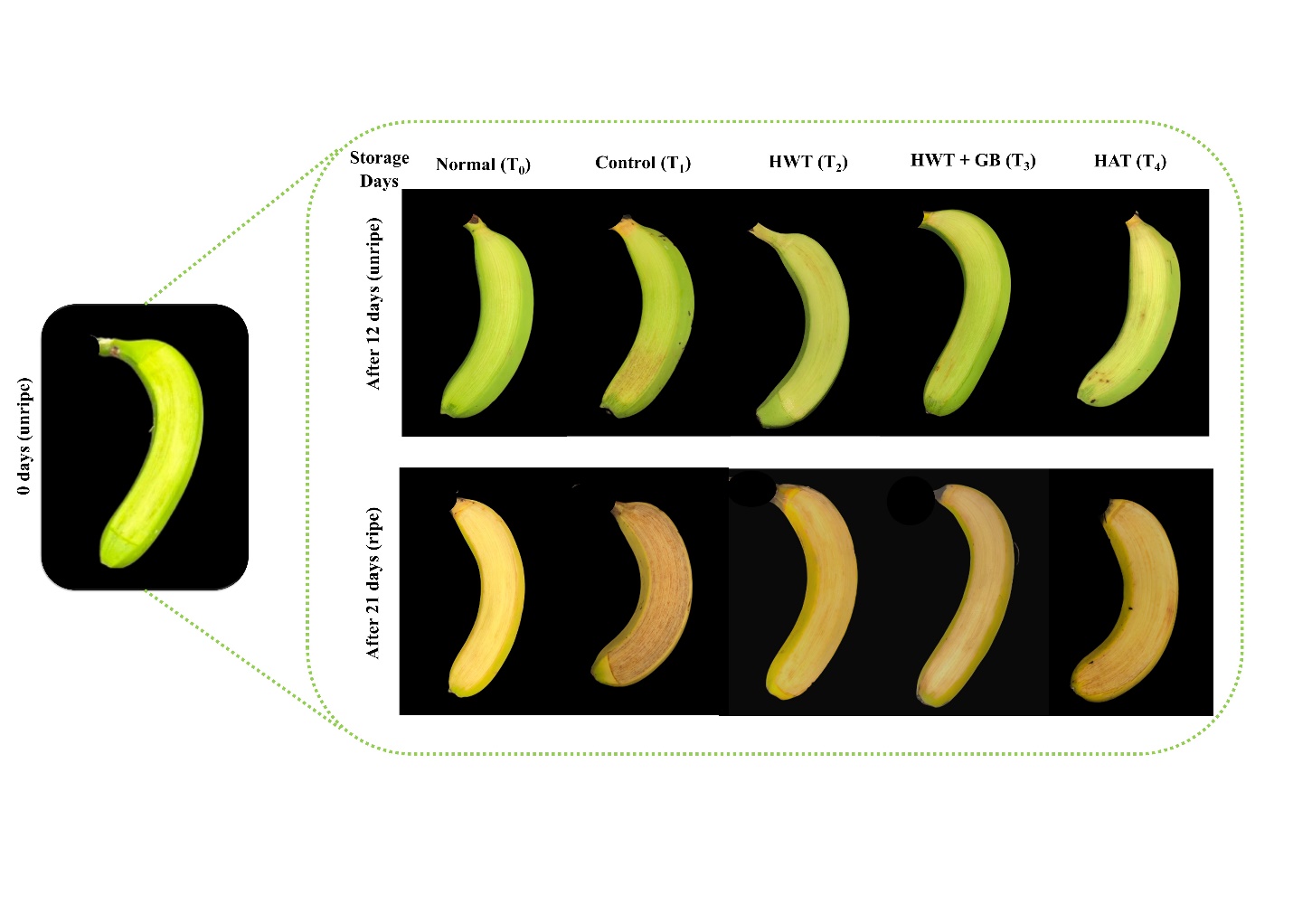


**Figure S1:** Phenotypic changes in bananas during storage and ripening under different postharvest treatments. Bananas were initially stored at 7 °C for 2 hours to induce chilling stress and then subjected to the following treatments: T_0_ (Normal: unchilled and untreated), T_1_ (Control: chilled, untreated), T_2_ (HWT: chilled, treated with hot water at 52 °C for 5 minutes), T_3_ (HWT+GB: chilled, treated with hot water at 52 °C for 5 minutes and 100 mM glycine betaine), and T_4_ (HAT: chilled, treated with hot air at 35 °C for 5 minutes). The images show banana fruit after 12 days of storage at 20 °C (unripe stage) and after 21 days, including ripening induced by 0.1% ethephon (ripe stage). Severe chilling injury symptoms (peel browning and discoloration) are visible in the control group (T_1_), while the treated groups, particularly HWT+GB (T_3_), exhibited reduced chilling injury and better visual quality.
